# Larger reductions in blood pressure during post‐exercise standing, but not middle cerebral artery blood velocity, in resistance‐trained versus untrained individuals

**DOI:** 10.1113/EP092327

**Published:** 2024-12-25

**Authors:** Stephanie Korad, Toby Mündel, Blake G. Perry

**Affiliations:** ^1^ School of Health Sciences Massey University Wellington New Zealand; ^2^ School of Sport, Exercise and Nutrition Massey University Palmerston North New Zealand; ^3^ Department of Kinesiology Brock University St Catharines Canada

**Keywords:** blood pressure, middle cerebral artery blood velocity, resistance exercise

## Abstract

Dynamic resistance exercise (RE) produces sinusoidal fluctuations in blood pressure, with hypotension and cerebral hypoperfusion commonly observed immediately following RE. Whether the cerebral vasculature adapts to these regular blood pressure challenges is unclear. This study examined the cerebrovascular response to post‐dynamic RE orthostasis. RE‐trained (*n* = 15, female = 4) and healthy untrained individuals (*n* = 15, female = 12) completed five stands: one after seated rest, with each of the subsequent four stands occurring immediately following a set of 10 repetitions of unilateral leg extension exercise at 60% of their one repetition maximum. Beat‐to‐beat blood pressure, mean middle cerebral artery blood velocity (MCAv_mean_) and end‐tidal carbon dioxide were measured throughout. During standing the mean arterial blood pressure (MAP) and MCAv_mean_ nadirs were identified. There was no difference between groups for age (mean ± SD, 26 ± 7 RE‐trained vs. 25 ± 6 years untrained, *P* = 0.683) or weight (78 ± 15 vs. 71 ± 15 kg, *P* = 0.683). At MAP nadir during the post‐exercise stand, a greater reduction in MAP was observed in the RE‐trained group (e.g., set 4, −45 ± 11 vs. −36 ± 6 mmHg, training effect *P* = 0.026). However, post‐exercise stand MCAv_mean_ at MCAv_mean_ nadir was not different (e.g., set 4, −20 ± 7 vs. −17 ± 6 cm/s, interaction effect *P* = 0.478). Rate of regulation was higher in the RE‐trained group (set 1, 0.301 ± 0.170 vs. 0.167 ± 0.009, training effect *P* = 0.023). Despite RE‐trained individuals demonstrating greater absolute reductions in MAP during orthostasis following RE, there were no differences in MCAv_mean_, suggesting that habitual RE may mitigate post‐exercise cerebral hypoperfusion.

## Abstract (highlights)

- **What is the central question of this study?**
  Resistance exercise produces vascular adaptations within the central arteries: does vascular adaptation also occur in the cerebral circulation?
- **What is the main finding and its importance?**
  Upon standing following moderate intensity dynamic resistance exercise, resistance‐trained individuals demonstrated a greater decrease in mean arterial pressure compared to untrained individuals. However, the middle cerebral artery blood velocity response did not differ between groups, which may indicate more effective cerebral autoregulation during acute reduction in blood pressure initiated by orthostasis in the resistance‐trained group.

## 1 INTRODUCTION

The benefits of resistance exercise (RE) have been well documented, with RE increasing muscle strength and muscle mass (Deschenes & Kraemer, 2002), improving mental health (O'Connor et al., 2010), decreasing fat mass (Lopez et al., 2022) and having a neuroprotective effect (Yarrow et al., 2010). However, despite the plethora of benefits associated with RE, RE can produce severe hypertension (MacDougall et al., 1985), with the increase in arterial blood pressure (ABP) during exercise dependent on the muscle mass recruited (MacDougall et al., 1992). The number of repetitions within a set (Sale et al., 1993), within succeeding sets of the same exercise (Libardi et al., 2017) and when rest periods between sets are reduced (Paulo et al., 2019) also contributes to increases in ABP. Despite the many benefits of RE, upon standing immediately following heavy RE, hypotension and cerebral hypoperfusion are evident (Moralez et al., 2012; Perry, Schlader et al., 2014; Romero & Cooke, 2007), with syncope reported following high intensity RE (Compton et al., 1973).

RE training has been observed to induce changes in blood vessel characteristics, with a decrease in compliance seen in the carotid artery after a single bout of high‐intensity RE (DeVan et al., 2005) and in RE‐trained individuals at rest (Miyachi, 2013; Miyachi et al., 2004), with increased arterial stiffness associated with elevated risk of cardiovascular disease (Mattace‐Raso et al., 2006; Mitchell et al., 2010). Furthermore, RE also increased indices of cerebrovascular resistance (Thomas et al., 2021), indicating that the vascular adaptations to RE may extend to the brain in RE‐trained individuals. Previously we (Korad et al., 2024) have reported that during dynamic RE at the same exercise intensity, RE‐trained individuals exhibited greater ABP during leg extension exercise versus their untrained counterparts. However, despite having significantly higher blood pressures, there was no difference in the within exercise mean middle cerebral artery blood velocity (MCAv_mean_), which suggests that the resistance‐trained individuals may have gained some functional adaptation to the repetitive exposure to the high blood pressures associated with resistance training. Whether these adaptations extend to the post‐exercise period when blood pressure is declining is not understood.

Previous studies investigating the effects of dynamic RE on the cerebrovascular response after standing predominately used a leg press machine (Moralez et al., 2012; Romero & Cooke, 2007) or upright squat (Perry, Schlader et al., 2014). The consensus of these studies was that a reduction in MCAv_mean_ was seen immediately during standing alongside an acute reduction in mean arterial blood pressure (MAP), with the reduction in MCAv_mean_ being exacerbated by pre‐exercise hyperventilation (Romero & Cooke, 2007) and dehydration (Moralez et al., 2012). Furthermore, Perry, Schlader et al. (2014) emphasised that higher relative loads produced larger post‐exercise hypotension that resulted in a proportionate reduction in MCAv_mean_. Transient hypertension is said to be buffered more effectively by cerebral autoregulation (CA) than hypotensive challenges – a concept termed hysteresis (Brassard et al., 2017; Roy et al., 2022; Tzeng et al., 2011). Roy et al. (2022) reported that resistance‐trained individuals did not exhibit signs of hysteresis during forced oscillations in ABP induced by repeated squat‐stands at a frequency of 0.10 Hz, but those in healthy sedentary and endurance trained groups did. Whether cerebrovascular function is modified in RE‐trained individuals during acute post‐exercise hypotension remains unknown.

Therefore, the purpose of this study was to examine the cerebrovascular response to orthostasis induced post‐RE hypotension in resistance‐trained and untrained individuals. We hypothesised that although resistance‐trained individuals would display a greater blood pressure response during RE there would be no difference in recovery indicating that RE‐trained individuals had better CA than the untrained.

## 2 METHODS

### 2.1 Ethics and informed consent

All participants were informed of the experimental procedures and aware of the purpose of this study, as well as the potential risks associated with participating. All participants provided written informed consent prior to partaking in the research. The study was approved by the Massey University Human Ethics Committee (SOA 21/22) and was in agreement with the latest version of the *Declaration of Helsinki* apart from registration in a database. These data were collected as part of a larger investigation exploring the cerebrovascular responses to RE. However, this study examined the cerebrovascular response to post‐RE stand and the resultant hypotension. Therefore, the baseline measures and the within‐exercise data from Korad et al. (2024) are complementary to this study.

### 2.2 Participants

An a priori power analysis (G*Power version 3.1.9.4; Heinrich Heine University Düsseldorf, Düsseldorf, Germany) was conducted using data from Moralez et al. (2012) with similar interventions (dynamic RE then stand), design and outcome measures (i.e., MCAv and MAP). Based on conventional α (0.05) and β (0.80) values, a minimum of 24 participants (*n* = 12 per training group) was required. A total of 30 participants (female = 16) were recruited for this study (pooled mean ± SD: age, 26 ± 6 years, height 175 ± 10 cm, weight 74 ± 15 kg, body mass index 24 ± 5 kg/m^2^), with 15 participants in each group (see Table 1 for training group anthropometric data). All participants were healthy and free of any medical conditions, were not taking any form of medication other than oral contraception (RE‐trained *n* = 1, untrained *n* = 3) or an intrauterine device (untrained = 1), were non‐smokers, and had no history or symptoms of cardiovascular, pulmonary, metabolic or neurological disease. Menstrual cycle phase was self‐reported by female participants with all visits occurring during the early follicular phase (low oestrogen and progesterone) and during the placebo phase for those using oral contraceptives. Korad et al. (2022) and Favre and Serrador (2019) have previously reported no differences in functional cerebrovascular responses to acute changes in MAP and CA between menstrual cycle phases. The participants also self‐reported their habitual exercise regimen to be assigned to RE‐trained individuals or healthy sedentary as described in our previous study (Korad et al., 2024).

**TABLE 1:** Participants anthropometric and strength measurements.

Variables	RE‐trained	Untrained	*P*
Sex (male:female)	11:4	3:12	N/A
Age (years)	26 ± 7	25 ± 6	0.683
Height (m)	1.77 ± .09	1.72 ± .09	0.167
Weight (kg)	78 ± 15	71 ± 15	0.683
BMI (kg/m^2^)	25 ± 4	24 ± 6	0.809
Leg extension predicted 1RM (kg)	76 ± 19	52 ± 15	**<0.001**
Leg extension 60% of 1RM (kg)	44 ± 12	30 ± 8	**<0.001**
RE experience (months)	49 ± 45	—	—

*Note*: Data are presented means ± SD. *n* = 30. *P*‐values shown in bold indicate statistical significance. Abbreviations: 1RM, one repetition maximum; BMI, body mass index; RE, resistance exercise.

### 2.3 Study design

All participants visited the temperature‐controlled laboratory twice, once initially for familiarisation and subsequently for the experimental session. A full explanation and demonstration of the risks of participation, and equipment and procedures utilised in the experiment were given during the familiarisation session. Upon providing consent, the middle cerebral artery (MCA) contralateral to the exercising limb was insonated for the measurement of MCAv_mean_ as described in the MCAv_mean_ section below. In addition, the participant's unilateral leg extension one repetition maximum (1RM) of the dominant leg was estimated using the Brzycki (1993) equation (Weight/[1.0278 − (0.0278 × Number of repetitions)]), and the working intensity for the trial, 60% of the 1RM (60%1RM), was calculated. The participant also practised executing the leg extension at 60%1RM whilst maintaining the requested pacing and breathing pattern outlined below.

### 2.4 Experimental protocol

**FIGURE 1:**
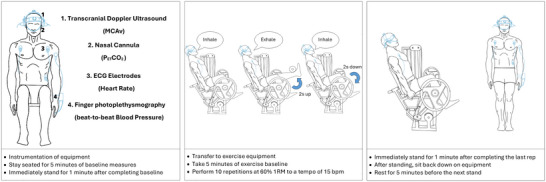
Experimental protocol. Initial baseline measurements were taken, then the pre‐exercise stand was performed for 1 min before sitting down on the leg extension machine. Pre‐exercise baseline measurements were taken during the 5‐min rest in between each exercise set. The exercise sets consisted of 10 repetitions of unilateral leg extensions followed by a 1‐min stand. The exercise sets consisted of 10 repetitions of unilateral leg extensions followed by a 1‐min stand. Haemodynamic variables (MCAv, blood pressure and heart rate) and partial pressure of end‐tidal carbon dioxide ($P_{\mathrm{ETC}O_{2}}$) were measured throughout.

The familiarisation occurred >1 week before the trial. Participants arrived at the laboratory having refrained from caffeinated beverages for 12 h and vigorous exercise and alcohol consumption for ≥24 h prior to testing. The participants were also instructed to consume 500 mL of water the night before and 500 mL approximately 4 h before the experiment to ensure euhydration (urine specific gravity (USG) < 1.020) (Sawka et al., 2009). The experimental overview is highlighted in Figure 1.

On arrival, a participant was asked to provide a urine sample for USG analysis. The participant was then seated on a chair for instrumentation. Once instrumented the participant rested quietly for 20 min for initial baseline recordings. Upon completion of baseline measures the participant stood up from the chair for 1 min for the pre‐exercise stand. Once the 1‐min stand was completed the participant was then transferred to the leg extension machine. Baseline values were continued for another 5 min, and immediately preceding each exercise set thereafter. During the exercise phase, the participant completed 10 repetitions (reps) of unilateral leg extensions at 60% of their 1RM. Pilot testing revealed that the loading was appropriate for untrained individuals to complete the exercise without fatigue or recruiting the Valsalva manoeuvre given (1) the number of repetitions and sets, (2) the length of rest periods, (3) the slow controlled contractions, and (4) the paced breathing (all described below). Furthermore, a moderate load decreased the risk of injury in an untrained cohort. The tempo was set to 15 reps/min, resulting in a repetition cycle length of 4 s, with 2 s given for both the concentric and eccentric phases as described by Korad et al. (2024). The breathing sequence was set to match the tempo of the exercise, with exhalation during the concentric phase (2 s) and inhalation during the eccentric phase (2 s). As such, all participants avoided the Valsalva manoeuvre during RE. Immediately following the completion of the 10 repetitions, the participant was instructed to stand and remain standing for 1 min. During the stand the participant was asked to remain standing quietly upright, with both feet parallel and on the floor and with as little movement as possible. If the participant felt dizzy or faint, they were instructed to sit down immediately. Once the 1‐min stand was completed the participant then sat back down on the leg extension machine and rested for 5 min while baseline measures were taken, before repeating the sequence again until a total of four sets of 10 reps were completed. Each participant was reminded of the breathing technique prior to each set, with the breathing and repetition timing aided by a metronome. The following criteria were used to ensure that the Valsalva manoeuvre was not performed:

1. No large and acute elevations in blood pressure were observed during exercise, beyond what would be expected for the prescribed intensity as indicated by previous repetitions. Previous studies reported that when the Valsalva manoeuvre is recruited during RE, MAP (but not necessarily MCAv) is acutely elevated within the repetition (Perry et al., 2020; Perry, Mündel et al., 2014).
2. Participants produced a capnograph that aligned with the paced breathing of the repetitions.
3. Participants were reminded of the exercise and breathing requirements for each set before and during each exercise bout.

### 2.5 Systemic haemodynamics

Heart rate (HR) was measured using a three‐lead electrocardiogram (ECG; ADInstruments, Bella Vista, NSW, Australia). Non‐invasive beat‐to‐beat ABP was measured by finger photoplethysmography (Finapres Medical Systems, Enschede, The Netherlands). The cuff was placed on the middle phalanx of either the middle finger or the index finger on the non‐dominant hand. The cuff was referenced to the level of the heart using the height correction unit. The middle panel of the schematic diagram in Figure 1 obscures the left hand, which for the purposes of this schematic diagram only, has the Finometer attached. The non‐dominant hand (with finger cuff attached) rested on the participant's lap, and the contralateral hand only was used to grip the leg‐extension machine. Blood pressure values were checked against an automated sphygmomanometer (Suresigns VM4, Philips Medical Systems, Best, The Netherlands) during baseline and 2 min following each exercise bout and corrected when necessary.

### 2.6 MCAv_mean_

Blood velocity in the contralateral middle cerebral artery to the exercising leg was measured during and following exercise using transcranial Doppler (TCD) ultrasonography (Doppler‐Box X, DWL, Compumedics, Singen, Germany). The contralateral MCA was selected as the motor tracts decussate. Thus, it is possible that neurovascular coupling, the matching of cerebral perfusion with local neuronal activity, would mediate a larger increase in the contralateral MCAv compared to the ipsilateral side, as seen in static handgrip exercise (Braz et al., 2014). Blood velocity in the M1 segment of the MCA was measured using a 2 MHz probe, fixed in position via an adjustable headband. The probe was fixed over the temporal window, above the zygomatic arch, using search techniques described elsewhere (Aaslid et al., 1989; Willie et al., 2011). Ultrasound gel (Tensive, Parker Laboratory, Fairfield, NY, USA) was placed between the transducer probe and the skin to obtain the highest quality image. The average depth of the insonated MCA in the current study was 54 ± 4 mm in alignment with Bathala et al. (2013).

### 2.7 Partial pressure of end‐tidal carbon dioxide

The partial pressure of end‐tidal carbon dioxide ($P_{\mathrm{ETC}O_{2}}$) was measured using an online gas analyser (ML206 Gas Analyser, ADInstruments) and was collected throughout using a nasal cannula. The gas analyser was calibrated to a known gas concentration before each experiment.

### 2.8 Urine analysis

Hydration status has been reported to influence cerebrovascular regulation (Moralez et al., 2012; Perry et al., 2016), and therefore USG was used to confirm hydration status before each experiment using a handheld refractometer (Atago Co., Ltd, Tokyo, Japan). All participants were instructed to consume 500 mL of water the night before and 500 mL approximately 4 h before the experiment. Approximately 30 min before the commencement of the experiment, USG was measured to confirm euhydration (mean ± SD 1.010 ± 0.007). If the participant's USG did not meet the required level of hydration, they were provided with approximately 500 mL of water. After consuming the water, the participant waited for 30 min before the USG was reassessed. This process was repeated until a USG value below 1.020 was achieved (Sawka et al., 2009).

### 2.9 Data acquisition

All data were collected continuously using an analog to digital converter (PowerLab, ADInstruments) interfaced with a computer and then analysed using LabChart software (v.8.1.13 ADInstruments).

### 2.10 Data analysis

Thirty participants were recruited for this study; however, only 28 participants had usable traces that could be analysed; one participant did not have usable traces for the post‐exercise stand data due to multiple premature ventricular contractions, and the MCAv traces were substandard during the post‐exercise stand for another participant. Therefore, the data presented moving herein will be for 28 participants, 14 participants in the RE‐trained group and 14 in the untrained group.

### 2.11 Dependent measures

MCAv_mean_ was calculated using the mean waveform of the raw MCAv trace. MAP was calculated using the formula 1/3 systolic blood pressure (SBP) + 2/3 diastolic blood pressure (DBP). Cerebrovascular conductance index (CVCi) was calculated using the equation CVCi = MCAv_mean_/MAP.

Baseline data were averaged during the last minute of rest before each set. In total, five stands were completed, with stand 1 occurring before exercise after the recording of baseline measures and stands 2–5 occurring immediately after each of the exercise sets. Pre‐stand measures were averaged for 4 s immediately before the stand, and as such for stands 2–5 (following sets one through four) these data represent the haemodynamic status during the last repetition of exercise. The MCAv_mean_ and MAP nadir were defined as the lowest values in a single cardiac cycle observed following the stand for each variable after which both began to recover (increase) toward baseline values. All key variables, including MCAv_mean_, systolic middle cerebral artery blood velocity (SMCAv), diastolic middle cerebral artery blood velocity (DMCAv), MAP, SBP, DBP, CVCi, HR and $P_{\mathrm{ETC}O_{2}}$, were measured at these nadir points. Furthermore, these values were used to calculate absolute changes from baseline (rest) and pre‐stand values (immediately prior to the stand). Furthermore, time to nadir was determined as the time taken to achieve the nadir values for both MAP and MCAv_mean_ at the onset of the stand. The time lag of these responses was also calculated as: time to MCAv_mean_ nadir – time to MAP nadir, as MCAv nadir is reached prior to MAP nadir following RE (Perry, Schlader et al., 2014).

The area under the curve (AUC) was calculated from a method previously described by Pruessner et al. (2003) and the AUC was calculated with respect to the baseline values. That is, the AUC calculation began when the variable of interest decreased below the baseline value and ended when the value then returned to the baseline value. The baseline value refers to the measurement taken during the resting period before each set. The time to recovery data were taken after the stand. Time to recovery was defined as the time it took from the MCAv or MAP values falling below baseline to when they returned to baseline, and was used to analyse time to recovery.

### 2.12 Rate of regulation

The rate of regulation (RoR) was used to evaluate the relationship between MAP and MCAv_mean_ during the acute hypotension induced by the stand immediately following RE. RoR was calculated using the method outlined by Labrecque et al. (2019), utilising the equation:

RoR=ΔCVCiΔCVCiΔtΔtΔMAP

where (ΔCVCi/Δ*t*) represents the linear regression slope of the change in cerebrovascular conductance index (CVCi) over time (*t*) during the initial regulatory response to standing (Aaslid et al., 1989; Ogoh et al., 2008). The time interval (Δ*t*) is a 2.5‐s period following the onset of the regulatory response, characterised by a continuous rise in CVCi upon standing (Labrecque et al., 2019). The change in MAP (ΔMAP) is calculated as the difference between 4 s immediately preceding the stand (Aaslid et al., 1989) and the average MAP during the initial haemodynamic response to standing, where changes in MCAv_mean_ occur independently of baroreflex control (van Beek et al., 2008).

%MCAv/%MAP was used to calculate the contribution of the MAP response to the MCAv reduction during each stand. The percentage change was calculated from the 4 s immediately prior to the stand to the MCA nadir following the stand.

### 2.13 Statistical analysis

All data were analysed using SPSS statistical software version 28 (IBM Corp., Armonk, NY, USA). Statistical significance was set at *P ≤ *0.05. Student's unpaired *t*‐test was performed to compare anthropometric, 1RM and 60%1RM data. A two‐way mixed ANOVA was performed to analyse baseline measures (training × baselines, 2 × 5) and dependent variables of interest during post‐dynamic RE stand (training × sets 2 × 5). *t*‐Tests were used for *post hoc* comparisons and a Bonferroni correction factor was used when necessary. Partial eta square (partial η^2^) is reported for the training by set interaction only, with large effect sizes identified as >0.1379, medium 0.0588–0.1379, and small <0.0099 (Cohen, 2013). All data are displayed as the mean ± SD.

## 3 RESULTS

Participants’ anthropometric and exercise measurements are presented in Table 1. There were no significant differences in the anthropometric measurements between the RE‐trained and untrained; however, RE‐trained had a greater predicted 1RM and 60% of 1RM versus their untrained counterparts (see Table 1 for *P*‐values). The RE‐trained group trained for 49 ± 45 months, ranging from 6 to 144 months of continuous RE training.

### 3.1 Baseline measurements

Baseline measures for the initial baseline immediately following instrumentation and baseline prior to each set were analysed. There were no significant main effects of training (all *P* ≥ 0.240) or training by set interaction (all *P* ≥ 0.111) for any baseline variables between groups. However, with the exception of HR and $P_{\mathrm{ETC}O_{2}}$, there was a set effect for all variables, whereby both training groups demonstrated equal ‘drift’ in the baseline variables (all *P* ≤ 0.009) whereby blood pressure variables (SBP, DBP and MAP) increased (e.g., MAP pre‐exercise: 82 ± 10 mmHg for RE‐trained and 81 ± 6 mmHg for untrained; vs. immediately prior to set 4: 90 ± 13 mmHg and 87 ± 8 mmHg; *P* < 0.001 for both *post hoc* tests).

### 3.2 Pre‐stand values measurements

There was no significant main effect of training for MCAv_mean_, SMCAv, DMCAv, CVCi, $P_{\mathrm{ETC}O_{2}}$ or HR (all *P* > 0.181; see Table 2 for values). However, a training by set interaction effect was observed for MAP (*P* = 0.002), SBP (*P* < 0.001) and DBP (*P* = 0.020). *Post hoc* tests revealed that the RE‐trained group had higher MAP in sets 2, 3 and 4 (all *P* < 0.006, Bonferroni corrected) and higher SBP in sets 3 and 4 (all *P* < 0.008, Bonferroni corrected). However, there were no *post hoc* difference for DBP observed (all *P* > 0.016, Bonferroni corrected) (see Table 2 for values). A set effect (*P* < 0.03) was seen for all variables, with MCAv_mean_, SMCAv, DMCAv, CVCi and $P_{\mathrm{ETC}O_{2}}$ decreasing across sets, while MAP, SBP, DBP and HR increased across sets (see Table 2 for values).

**TABLE 2:** Resistance‐trained versus untrained pre‐stand values for cerebrovascular and cardiovascular measures.

Variable	Condition	Sets					*P*			Partial ƞ^2^
		Pre‐exercise	Set 1	Set 2	Set 3	Set 4	Training	Set	Interaction	
MCAv_mean_ (cm/s)	RE‐trained	69 ± 11	64 ± 12^a^	64 ± 14^a^	64 ± 13	65 ± 13	0.766	0.001	0.074	0.078
	Untrained	70 ± 13	69 ± 13^a^	67 ± 12^a^	65 ± 10^b^	62 ± 11^abcd^				
SMCAv_mean_ (cm/s)	RE‐trained	103 ± 16	98 ± 18^a^	94 ± 18^a^	94 ± 17^a^	96 ± 17^a^	0.630	<0.001	0.167	0.060
	Untrained	106 ± 20	102 ± 18^a^	100 ± 19^a^	98 ± 16^ab^	93 ± 16^abcd^				
DMCAv_mean_ (cm/s)	RE‐trained	50 ± 8	46 ± 10^a^	46 ± 10^a^	46 ± 10	48 ± 10^c^	0.940	0.003	0.058	0.083
	Untrained	50 ± 9	49 ± 10^a^	47 ± 9^a^	46 ± 8^a^	44 ± 8^abcd^				
CVCi (cm/s/mmHg)	RE‐trained	0.80 ± 0.18	0.63 ± 0.14^a^	0.61 ± 0.18^a^	0.60 ± 0.18^a^	0.60 ± 0.17^ab^	0.242	<0.001	0.534	0.029
	Untrained	0.85 ± 0.16	0.70 ± 0.14^a^	0.69 ± 0.13^a^	0.68 ± 0.11^a^	0.64 ± 0.12^abcd^				
MAP (mmHg)	RE‐trained	87 ± 10	104 ± 11^ab^	108 ± 12*^ab^	110 ± 14*^ab^	112 ± 14*^abc^	0.012	<0.001	0.002	0.312
	Untrained	83 ± 7	100 ± 11^a^	97 ± 8^a^	96 ± 9 ^a^	98 ± 8^a^				
SBP (mmHg)	RE‐trained	127 ± 16	149 ± 22^a^	154 ± 23^a^	156 ± 28*^a^	159 ± 29*^ab^	0.020	<0.001	<0.001	0.175
	Untrained	120 ± 12	138 ± 21^a^	134 ± 16^a^	132 ± 16^a^	133 ± 16^a^				
DBP (mmHg)	RE‐trained	68 ± 9	81 ± 8^a^	85 ± 8^ab^	87 ± 10^ab^	88 ± 9^ab^	0.050	<0.001	0.030	0.097
	Untrained	65 ± 7	80 ± 9^a^	78 ± 8^a^	78 ± 10^a^	80 ± 7^a^				
$P_{\mathrm{ETC}O_{2}}$ (mmHg)	RE‐trained	38 ± 5	37 ± 4^a^	35 ± 4^a^	36 ± 4^a^	36 ± 4^a^	0.181	<0.001	0.640	0.024
	Untrained	37 ± 5	34 ± 4^a^	34 ± 4^a^	33 ± 3^a^	33 ± 4^a^				
HR (bpm)	RE‐trained	73 ± 18	86 ± 27^a^	89 ± 16^a^	90 ± 14^a^	95 ± 21^a^	0.602	<0.001	0.498	0.032
	Untrained	71 ± 15	93 ± 13^a^	94 ± 14^a^	95 ± 13^a^	94 ± 13^a^				

*Note*: Data are presented means ± SD. RE‐trained, *n* *= *14; untrained; *n* = 14. Despite a significant training by set interaction for DBP, *post hoc* tests revealed no differences between training groups (*P* ≥ 0.016, Bonferroni corrected); however, *post hoc* test revealed significant differences for MAP (all *P* ≤ 0.006, Bonferroni corrected) and SBP (all *P* ≤ 0.008, Bonferroni corrected); *differences between groups within a set. ^a^Different from initial baseline. ^b^Different from set 1. ^c^Different from set 2. ^d^Different from set 3. Abbreviations: CVCi, cerebrovascular conductance index; DMCAv, diastolic middle cerebral artery blood velocity; HR; heart rate; MAP, mean arterial blood pressure; MCAv_mean_, mean middle cerebral artery blood velocity; $P_{\mathrm{ETC}O_{2}}$, end‐tidal partial pressure of carbon dioxide; RE‐trained, resistance‐trained; SMCAv, systolic middle cerebral artery blood velocity.

### 3.3 Reduction from baseline to MCAv_mean_ nadir

The baseline to MCAv_mean_ nadir cerebrovascular variables reported set differences for all variables barring SMCAv (*P* = 0.213) and $P_{\mathrm{ETC}O_{2}}$ (*P* = 0.616). The set differences seen in MCAv_mean_, DMCAv, CVCi and HR were observed to increase in magnitude from baseline from pre‐exercise measures to set 4 (set effect; all *P* < 0.042), whilst MAP, SBP and DBP were observed to have decreased across the sets (all *P* < 0.001).

### 3.4 Absolute reduction from pre‐stand values to MCAv_mean_ nadir

A training effect was observed for MAP (*P* = 0.036), DBP (*P* = 0.016) and HR (*P* = 0.048), with the RE‐trained group exhibiting greater reductions for all three variables (see Table 3 for values). Set differences were observed for SMCAv, MAP, DBP, $P_{\mathrm{ETC}O_{2}}$ and HR (*P* ≤ 0.011) for the pre‐stand values to MCAv_mean_ nadir (see Table 3 for values). HR difference across the sets was the only variable that decreased in magnitude across the sets (all *P* < 0.001), while the rest of the variables increased in difference from pre‐exercise to set 4 (all *P* < 0.005).

**TABLE 3:** Resistance‐trained versus untrained absolute change from pre‐stand values to MCAv_mean_ nadir.

Variable	Condition	Sets					*P*			Partial *ƞ* ^2^
		Pre‐exercise	Set 1	Set 2	Set 3	Set 4	Training	Set	Interaction	
MCAv_mean_ (cm/s)	RE‐trained	−20 ± 8	−16 ± 6^a^	−18 ± 5^a^	−19 ± 7	−20 ± 7	0.752	0.647	0.478	0.033
	Untrained	−19 ± 8	−18 ± 7^a^	−18 ± 6^a^	−18 ± 7	−17 ± 6				
SMCAv_mean_ (cm/s)	RE‐trained	−4 ± 10	15 ± 20^a^	11 ± 6^a^	11 ± 10^a^	9 ± 11^a^	0.225	<0.001	0.229	0.052
	Untrained	−4 ± 9	3 ± 13^a^	7 ± 11^a^	10 ± 18^a^	6 ± 9^a^				
DMCAv_mean_ (cm/s)	RE‐trained	−23 ± 12	−22 ± 9^a^	−25 ± 8^a^	−25 ± 9	−28 ± 8^b^	0.127	0.195	0.908	0.010
	Untrained	−21 ± 11	−19 ± 7^a^	−21 ± 9^a^	−22 ± 10	−22 ± 8				
CVCi (cm/s/mmHg)	RE‐trained	−0.07 ± 0.15	−0.07 ± 0.11^a^	−0.06 ± 0.07^a^	−0.05 ± 0.11	−0.05 ± 0.08	0.717	0.938	0.967	0.005
	Untrained	−0.05 ± 0.10	−0.06 ± 0.12^a^	−0.07 ± 0.12^a^	0.04 ± 0.09	0.05 ± 0.08				
MAP (mmHg)	RE‐trained	−30 ± 12	−33 ± 6^a^	−36 ± 7^a^	−38 ± 10	−39 ± 10^ab^	0.036	0.011	0.569	0.028
	Untrained	−26 ± 10	−31 ± 10^a^	−31 ± 7^a^	−30 ± 10	−31 ± 9				
SBP (mmHg)	RE‐trained	−33 ± 21	−31 ± 10^a^	−34 ± 12^a^	−37 ± 16	−39 ± 19	0.133	0.884	0.567	0.028
	Untrained	−28 ± 14	−30 ± 17^a^	−29 ± 11^a^	−28 ± 14	−28 ± 14				
DBP (mmHg)	RE‐trained	−29 ± 9	−34 ± 5^a^	−36 ± 5^a^	−38 ± 8^a^	−39 ± 7^ab^	0.016	<0.001	0.657	0.023
	Untrained	−24 ± 10	−32 ± 8^a^	−33 ± 5^a^	−31 ± 9	−32 ± 7 ^a^				
$P_{\mathrm{ETC}O_{2}}$ (mmHg)	RE‐trained	−1 ± 2	3 ± 2^a^	3 ± 3^a^	3 ± 3^a^	1 ± 8	0.755	0.005	0.328	0.043
	Untrained	−0 ± 2	2 ± 3^a^	3 ± 3^a^	2 ± 2^a^	2 ± 2^a^				
HR (bpm)	RE‐trained	25 ± 15	15 ± 24^a^	12 ± 21^a^	4 ± 30^a^	6 ± 22^a^	0.048	<0.001	0.627	0.024
	Untrained	16 ± 10	1 ± 7^a^	−1 ± 5^a^	1 ± 5^a^	−1 ± 6^a^				

*Note*: Data are presented means ± SD. RE, trained, *n* *= *14; untrained, *n* = 14. ^a^Different from initial baseline. ^b^Different from set 1. Abbreviations: CVCi, cerebrovascular conductance index; DMCAv, diastolic middle cerebral artery blood velocity; HR; heart rate; MAP; mean arterial blood pressure; MCAv_mean_, mean middle cerebral artery blood velocity; $P_{\mathrm{ETC}O_{2}}$, end‐tidal partial pressure of carbon dioxide; RE, trained, resistance‐trained; SMCAv, systolic middle cerebral artery blood velocity.

### 3.5 Reduction from baseline to MAP nadir

Set differences were observed for SMCAv, MAP and DBP for baseline to MAP nadir data. SMCAv differences increased from pre‐exercise to set 4 (*P* < 0.001; SMCAv pre‐exercise: −4 ± 19 mmHg RE‐trained, −4 ± 8 mmHg untrained; vs. set 4: 4 ± 22 mmHg RE‐trained, −1 ± 9 mmHg untrained). MAP and DBP differences decreased across the sets (all *P* ≤ 0.024; SBP pre‐exercise: −29 ± 17 mmHg RE‐trained, −27 ± 14 mmHg untrained; vs. set 4: −19 ± 23 mmHg RE‐trained, −25 ± 17 mmHg untrained).

### 3.6 Reduction from pre‐stand to MAP nadir

Training effects were observed for MAP (*P* = 0.026) and DBP (*P* = 0.010), with the resistance‐trained group having higher differential values for both variables (see Table 4 for values). All variables except for DMCAv and SBP demonstrated set differences with decreases across sets being seen for MCAv_mean_, SMCAv, CVCi and HR (all *P* ≤ 0.004, see Table 4 for values). MAP, DBP and $P_{\mathrm{ETC}O_{2}}$ showed increases across sets (all *P* < 0.001).

**TABLE 4:** Resistance‐trained versus untrained absolute change from pre‐stand values to MAP nadir.

Variable	Condition	Sets					*P*			Partial ƞ^2^
		Pre‐exercise	Set 1	Set 2	Set 3	Set 4	Training	Set	Interaction	
MCAv_mean_ (cm/s)	RE‐trained	−18 ± 7	−9 ± 7^a^	−11 ± 8^a^	−13 ± 9	−13 ± 11	0.630	0.001	0.321	0.044
	Untrained	−17 ± 7	−13 ± 9^a^	−14 ± 8^a^	−14 ± 9	−12 ± 5^a^				
SMCAv_mean_ (cm/s)	RE‐trained	−4 ± 10	16 ± 14^a^	18 ± 8^a^	17 ± 13^a^	14 ± 12^a^	0.235	<0.001	0.184	0.057
	Untrained	−4 ± 9	9 ± 13^a^	15 ± 16^a^	8 ± 10^a^	12 ± 9^a^				
DMCAv_mean_ (cm/s)	Re‐trained	−23 ± 12	−16 ± 9^a^	−19 ± 8^a^	−22 ± 9	−19 ± 10	0.598	0.318	0.493	0.032
	Untrained	−18 ± 9	−18 ± 8^a^	−19 ± 10^a^	−19 ± 10	−19 ± 9				
CVCi (cm/s/mmHg)	RE‐trained	−0.14 ± 0.12	−0.23 ± 0.13^a^	−0.22 ± 0.11^a^	−0.19 ± 0.10	−0.20 ± 0.11	0.652	0.004	0.878	0.011
	Untrained	−0.12 ± 0.08	−0.21 ± 0.12^a^	−0.19 ± 0.11^a^	0.19 ± 0.17	0.21 ± 0.16				
MAP (mmHg)	RE‐trained	−33 ± 11	−38 ± 7^ab^	−43 ± 8^ab^	−44 ± 9^ab^	−45 ± 11^ab^	0.026	<0.001	0.092	0.073
	Untrained	−28 ± 9	−38 ± 10^a^	−36 ± 6^a^	−35 ± 10^a^	−36 ± 8^a^				
SBP (mmHg)	RE‐trained	−38 ± 20	−40 ± 11^a^	−45 ± 13^a^	−46 ± 16^b^	−49 ± 22^b^	0.101	0.058	0.097	0.072
	Untrained	−32 ± 12	−41 ± 14^a^	−35 ± 10^a^	−35 ± 16	−36 ± 14				
DBP (mmHg)	RE‐trained	−30 ± 8	−37 ± 5^ab^	−42 ± 5^ab^	−43 ± 7^ab^	−43 ± 7^ab^	0.010	<0.001	0.180	0.058
	Untrained	−26 ± 8	−36 ± 7^a^	−35 ± 9^a^	−35 ± 9^a^	−36 ± 6^a^				
$P_{\mathrm{ETC}O_{2}}$ (mmHg)	RE‐trained	−1 ± 3	3 ± 2^a^	4 ± 2^a^	4 ± 3^a^	3 ± 2^a^	0.359	<0.001	0.416	0.037
	Untrained	−0 ± 2	3 ± 3^a^	4 ± 3^a^	3 ± 3^a^	2 ± 2^a^				
HR (bpm)	RE‐trained	21 ± 26	11 ± 20^a^	−8 ± 37^a^	13 ± 20^c^	−6 ± 9^a^	0.729	<0.001	0.219	0.053
	Untrained	19 ± 11	2 ± 7^a^	1 ± 7^a^	2 ± 7^a^	2 ± 9 ^a^				

*Note*: Data are presented means ± SD. RE‐trained, *n = *14; untrained, *n *= 14. ^a^Different from initial baseline. ^b^Different from set 1. Abbreviations: CVCi, cerebrovascular conductance index; DMCAv, diastolic middle cerebral artery blood velocity; HR, heart rate; MAP, mean arterial blood pressure; MCAv_mean_, mean middle cerebral artery blood velocity; $P_{\mathrm{ETC}O_{2}}$, end‐tidal partial pressure of carbon dioxide; RE‐trained, resistance‐trained; SMCAv, systolic middle cerebral artery blood velocity.

### 3.7 Haemodynamic recovery during stand

**FIGURE 2:**
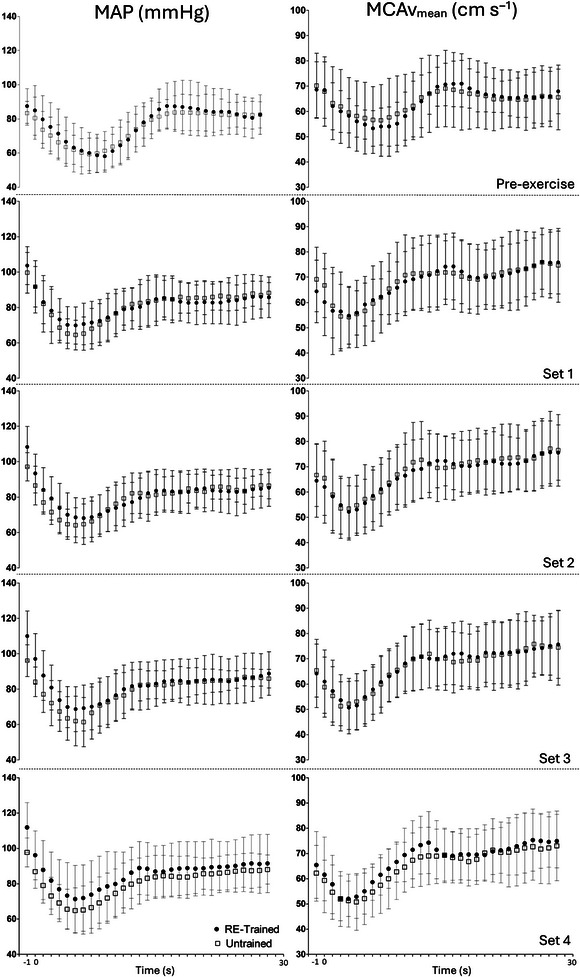
Temporal changes in mean arterial pressure (MAP) and mean middle cerebral artery blood velocity (MCAv_mean_) during recovery from standing. On the *x*‐axes, −1 indicates the mean values for the 4 s prior to standing, which includes the last repetition of RE (i.e., pre‐stand values) and standing occurred at time point 0. RE‐trained (resistance exercise‐trained), *n* = 14; untrained, *n* = 14.

There were no significant differences between the RE‐trained and untrained groups for MCAv_mean_ (interaction effect *P* = 0.657; RE‐trained: pre‐exercise: 14 ± 7 s; set 1: 13 ± 9 s; set 2: 12 ± 6 s; set 3: 12 ± 7 s; set 4: 11 ± 6 s; vs. untrained group means: pre‐exercise: 17 ± 18 s; set 1: 13 ± 7 s; set 2: 12 ± 6 s; set 3: 13 ± 12 s; set 4: 15 ± 9 s) and MAP (interaction effect *P* = 0.810; RE‐trained: pre‐exercise: 17 ± 8 s; set 1: 17 ± 10 s; set 2: 21 ± 12 s; set 3: 17 ± 9 s; set 4: 19 ± 11 s; versus untrained group: pre‐exercise: 16 ± 9 s; set 1: 17 ± 11 s; set 2: 21 ± 19 s; set 3: 19 ± 19 s; set 4: 22 ± 19 s) time to recovery after the stand. As illustrated in Figure 2, no significant differences were seen between the RE‐trained and untrained group during recovery. Similarly, the AUC analysis for MCAv_mean_ and MAP were also not different between groups (MCAv_mean_: interaction effect *P* = 0.428; RE‐trained: pre‐exercise: −139 ± 66; set 1: −147 ± 138; set 2: −129 ± 68; set 3: −123 ± 70; set 4: −115 ± 65; vs untrained: pre‐exercise: −172 ± 253; set 1: −149 ± 109; set 2: −139 ± 80; set 3 −154 ± 133; set 4: −220 ± 251; and MAP: interaction effect *P* = 0.982; RE‐trained: pre‐exercise: −236 ± 86; set 1: −203 ± 143; set 2: −265 ± 237; set 3: −203 ± 155; set 4: −252 ± 198; vs untrained: pre‐exercise: −242 ± 131; set 1: −204 ± 156; set 2: −259 ± 268; set 3: −227 ± 200; set 4: −281 ± 196).

### 3.8 Time to nadir

RE‐trained and untrained individuals showed no significant differences in MCAv_mean_ nadir (interaction *P* = 0.452) and MAP time to nadir (*P* = 0.818). Furthermore, there were no significant differences observed in the time‐lag difference between the groups (*P* = 0.592).

### 3.9 Cerebral autoregulation

A significant effect of training only was observed in RoR (*P* = 0.023), with the RE‐trained group having a greater RoR (pre‐exercise: 0.215 ± 0.12; set 1: 0.301 ± 0.17; set 2: 0.312 ± 0.10; set 3: 0.280 ± 0.12; set 4: 0.278 ± 0.15) versus the untrained group (pre‐exercise: 0.172 ± 0.14; set 1: 0.167 ± 0.09; set 2: 0.223 ± 0.13; set 3: 0.240 ± 0.10; set 4: 0.229 ± 0.08). However, there were no significant training (*P* = 0.973), set (*P* = 0.384), or training by set (*P* = 0.959) effects between groups for the ratio of percentage change in MCAv versus percentage change in MAP at MCAv nadir.

## 4 DISCUSSION

The purpose of this study was to investigate the effects of training status on the cerebrovascular response to post‐RE hypotension. The main findings from this study were as follows. (1) The RE‐trained group had greater MAP pre‐stand values than their untrained counterparts – indicating higher MAP in the last repetitions of RE. (2) The RE‐trained individuals demonstrated a larger decrease in MAP upon standing post‐dynamic RE compared to the untrained individuals. However, (3) despite the greater reduction of MAP following RE in the RE‐trained group, there was no group difference in the MCAv_mean_ response. Furthermore, this was supported by (4) the recovery time and AUC being not different between the groups and (5) the RE‐trained individuals displaying significantly greater RoR values suggesting a more effective CA in the RE‐trained group. Collectively, these data indicate that RE‐trained individuals are more effective in countering the orthostasis induced post‐exercise hypotension as there was no difference in MCAv_mean_, indicating more efficient CA, which is in accordance with our hypothesis.

Despite larger absolute decreases in MAP for RE‐trained individuals, both groups experienced a smaller reduction in MAP post‐stand than reported by others. Previous studies, using bilateral leg press exercises at higher intensities (>65% of 1RM) (Moralez et al., 2012; Romero & Cooke, 2007) reported greater MAP reductions (>70 mmHg), likely due to larger engaged muscle mass and higher exercise intensity. As within exercise MAP responses are muscle mass and intensity dependent, the within‐exercise MAP begins at a greater value (MacDougall et al., 1992) and has further to ‘fall’ to achieve the MAP nadir value and return to baseline. Due to the use of unilateral leg extension at the moderate intensity of 60% 1RM in the current study, smaller post‐RE MAP decreases are reported herein, aligning with Perry, Schlader et al. (2014), who noted that higher exercise intensities produce a greater rise in MAP during exercise and increases the magnitude of post‐exercise hypotension.

Despite a greater decrease in MAP in the RE‐trained group during the stand following dynamic RE, the reduction in MCAv_mean_ did not differ between training groups. This aligns with our previous analysis of the current data, indicating that despite greater within‐exercise MAP perturbations in the RE trained compared to the untrained group, the MCAv_mean_ response was similar between groups (Korad et al., 2024). It is therefore possible that the RE‐trained group have improved autoregulation. Indeed, RoR was greater in the RE‐trained group versus the untrained in all stands. Additionally, analysis of the AUC and time to recovery data revealed no group differences, indicating that the magnitude (extent of the decrease and duration) of the post‐RE hypotension and cerebral hypoperfusion was similar in the context of this experiment. Despite RE‐trained individuals experiencing a larger initial drop in MAP, their CA effectively compensated, allowing them to return to baseline MAP values just as quickly as the untrained group. This is noteworthy, as greater reductions in MAP are associated with increased symptoms of orthostatic instability (Romero & Cooke, 2007). Although Perry et al. (2019) found no difference in CA between RE‐trained, aerobic‐trained, and untrained individuals, further analysis of these data by Roy et al. (2022) indicated that the directional sensitivity of CA is present in sedentary, but not RE trained, individuals. That is, when comparing CA effectiveness during bidirectional ABP perturbations, untrained individuals demonstrated a greater ability to defend against increases in ABP during repeated squat–stands at both 0.10 and 0.05 Hz frequencies. Collectively, these data indicate that habitual RE may produce subtle differences in CA, although comparing studies is difficult due to the different methods used to measure CA (Brassard et al., 2024).

The partial pressure of arterial CO_2_ content ($P_{\mathrm{aC}O_{2}}$) is a potent regulator of cerebral blood flow (CBF), with elevations in $P_{\mathrm{aC}O_{2}}$ increasing CBF, whilst a reduction has the opposite response (Ainslie & Duffin, 2009). Whilst MAP rapidly fluctuates during RE, the overriding influence of $P_{\mathrm{aC}O_{2}}$ persists, with pre‐RE hyperventilation reducing MCAv_mean_ during leg press exercise (Romero & Cooke, 2007). Furthermore, increases in MCAv_mean_ are seen during fatiguing isometric handgrip exercise only when $P_{\mathrm{ETC}O_{2}}$ (as a proxy for arterial CO_2_ content) is clamped at near baseline levels (Braz et al., 2014). Importantly for the current study, there was no observed difference in $P_{\mathrm{ETC}O_{2}}$ between groups at pre‐stand values (i.e., end of exercise) and at MCAv_mean_ or MAP nadir, which suggests that the MCAv_mean_ responses were unlikely to be influenced by $P_{\mathrm{ETC}O_{2}}$. However, although there are no statistical differences during or following RE in the current study, given the potency of CBF regulation by $P_{\mathrm{aC}O_{2}}$ even small reductions in $P_{\mathrm{aC}O_{2}}$ induced by the paced breathing during RE in the current experiment should be considered when interpreting the data.

The repetitive exposure to high intensity (∼80% of 1RM) sinusoidal fluctuations in ABP has been observed to elicit adverse adaptations within the cardiorespiratory system, with a reduction in central arterial compliance (DeVan et al., 2005; Miyachi, 2013; Miyachi et al., 2004; Okamoto et al., 2009; Palmiere et al., 2018) and increased cerebrovascular resistance at rest when compared to sedentary controls (Nakamura & Muraoka, 2018; Thomas et al., 2021), although the last point is not a consistent finding (Corkery et al., 2021). Koch et al. (2005) reported that immediately after (<90 s) RE, CA was temporarily impaired in RE‐trained individuals. Furthermore, dynamic RE elicits an intensity‐dependent rise in blood flow turbulence and endothelial shear stress in the brain and the common carotid artery (Montalvo et al., 2022) and increased anterograde shear rate and blood flow in inactive limbs (Thomas et al., 2020). These findings suggest that dynamic RE significantly elevates shear rate and endothelial shear stress, which are accompanied by sinusoidal fluctuations in cerebral blood flow and perfusion pressure, potentially leading to acute changes in cerebrovascular function (Dawson et al., 2021).

### 4.1 Limitations

The mechanisms underpinning CA are not yet fully understood; however, it may be that large sinusoidal fluctuations in MAP are needed to engage the functional adaptations of RE as challenges at lower frequencies are effectively buffered by CA as previously elucidated by Perry and Lucas (2021). The current study shows that standing after moderate intensity dynamic RE induces large absolute decreases in ABP in RE‐trained individuals; however, the dynamic RE was unilateral, engaging a modest muscle mass and therefore inducing moderate increases in blood pressures. Confirmation of these data at higher intensities, or RE recruiting a greater muscle mass is required.

It is important to acknowledge certain methodological constraints when interpreting the results. Our study utilised the Transcranial Doppler Ultrasound as a non‐invasive way to measure MCAv_mean_ as a proxy for cerebral blood flow. Though the TCD provided continuous, real‐time measurements, the accuracy of MCAv as a proxy for cerebral blood flow relies on the assumption that the diameter of the middle cerebral artery being measured remains constant (Ainslie & Hoiland, 2014). Previous research has shown that MCA diameter may change under various physiological conditions. Verbree et al. (2014) found no significant alterations in MCA diameter during mild hypocapnia (a 7.5 mmHg reduction in end‐tidal CO_2_ or $P_{\mathrm{ETC}O_{2}}$). However, Coverdale et al. (2014) observed that the actual decrease in cerebral blood flow during more severe hypocapnia ($P_{\mathrm{ETC}O_{2}}$∼23 mmHg) was 7 ± 4% greater than the TCD‐measured change in MCAv. Decreases in $P_{\mathrm{ETC}O_{2}}$ (∼1 to 2 mmHg) were observed in the baseline and pre‐stand measures and this is because of the implementation of paced breathing during the dynamic RE. The mild hypocapnic stimulus in the current study is unlikely to have generated a significant change in MCA diameter. However, Verbree et al. (2017), using high‐resolution magnetic resonance imaging, reported a 2% decrease in MCA cross‐section during simple handgrip exercise, potentially due to sympathetic vasoconstriction. Given these considerations, it is possible that the RE protocol used before the stand may have induced some degree of MCA vasoconstriction. Therefore, the findings of the current study should be interpreted with caution.

Both females and males participated in the current study. Male participants typically demonstrate a more pronounced exercise pressor response, with greater increases in ABP during static handgrip exercise compared to females (Ettinger et al., 1996; Matthews & Stoney, 1988; Simoes et al., 2013). However, previous research examining sex differences in haemodynamic responses to dynamic exercise reported that when body surface area, composition (Bassareo & Crisafulli, 2020), body size and strength are comparable between sexes (i.e., no statistical differences), these differences in exercise pressor reflex become minimal or non‐existent (Tharpe et al., 2023). There were no group differences in the anthropometric measures in the current study, and furthermore, the cardiovascular measures were not different during any of the baseline period. Additionally, a meta‐analysis by Skinner et al. (2021) and subsequently a more recent study by Korad et al. (2022) reported no difference in CA across the menstrual cycle phases.

Though this study included both RE‐trained and untrained groups, a higher proportion of females participated in the untrained group compared to the RE‐trained group. Whilst this imbalance was not intentional, we chose to include these data given the limited research in this area with female participants. There is some evidence to indicate that there are sex differences in autoregulation and haemodynamic responses to forced perturbations in blood pressure. However, the results of these studies indicate superior cerebral autoregulation in females. During a supine–sit–stand protocol Abidi et al. (2017) reported lower MCAv_mean_ in males. In the same study, Abidi et al. reported that during the recovery of blood pressure during late phase II of the Valsalva manoeuvre, MCAv_mean_ was greater in eumenorrhoeic females during the high hormone phase of the menstrual cycle (luteal phase) compared to males. Similarly, during the same phase of the Valsalva manoeuvre, females using oral contraceptive (during both placebo and hormone phase) exhibited significantly greater diastolic MCAv compared to males. Moreover, Favre and Serrador (2019) reported significantly improved cerebral autoregulation in females (transfer function analysis during repeated squat stands) and females had a smaller decrease in MCAv_mean_ during sit‐to‐stand manoeuvres. When considering the regulation of blood pressure, females demonstrate greater baroreflex sensitivity compared to males during acute reductions in blood pressure (Fu et al., 2009). Collectively, these data indicate superior haemodynamic counter‐regulation during acute perturbations in blood pressure of females compared to males. Given the greater number of females in the untrained group, it would therefore be expected that the untrained group would have superior cerebral autoregulation and blood pressure regulation during the acute reduction in blood pressure induced by the experimental conditions of the current study. However, this was not evident, thus supporting our findings that the difference between groups is due to habitual exercise.

### 4.2 Conclusion

The results of the current study indicate that despite the RE‐trained group demonstrating a greater absolute decrease in post‐RE blood pressure compared to the untrained group, the MCAv_mean_ response was similar between groups This indicates that the RE‐trained group has a more efficient cerebral autoregulation and it may be possible that habitual RE could induce functional vascular adaptations that assists with maintaining cerebral blood flow during more pronounced blood pressure reductions. Future studies are required to corroborate the current findings at rest and following more intense exercise with a larger muscle mass that would produce a more pronounced challenge to cerebral autoregulation.

## AUTHOR CONTRIBUTIONS

Stephanie Korad, Toby Mündel and Blake Perry, conceptualised and design the research. Stephanie Korad and Blake Perry were responsible for data collection. Stephanie Korad, Toby Mündel and Blake Perry were responsible for data analysis, interpretation and drafting of the article. All authors have read and reviewed the article and provided critical feedback. All authors have approved the final version of this manuscript and agree to be accountable for all aspects of the work in ensuring that questions related to the accuracy or integrity of any part of the work are appropriately investigated and resolved. All persons designated as authors qualify for authorship, and all those who qualify for authorship are listed.

## CONFLICT OF INTEREST

The authors declare they have no conflicts of interest.

## Data Availability

The data that support the findings of this study are available from the corresponding author upon reasonable request.
